# Pain and haemorrhage are the most common reasons for emergency department use and hospital admission in adults following ambulatory surgery: results of a population-based cohort study

**DOI:** 10.1186/s13741-020-00155-3

**Published:** 2020-08-19

**Authors:** Monakshi Sawhney, David H. Goldstein, Xuejiao Wei, Genevieve C. Pare, Louie Wang, Elizabeth G. VanDenKerkhof

**Affiliations:** 1 Department of Anesthesiology, University of Calgary, South Health, Campus, 4448 Front Street SE, Calgary, Alberta T3M 1M4 Canada; 2 Institute for Clinical Evaluative Sciences, Queen’s University, 21 Arch Street, Suite 208, Kingston, Ontario K7L 3N6 Canada; 3 School of Nursing, Queen’s University, 92 Barrie Street, Kingston, Ontario K7L 3N6 Canada; 4 Department of Anesthesiology and Perioperative Medicine, Queen’s University, Victory 2, Kingston General Hospital, 76 Stuart Street, Kingston, Ontario K7L 2V7 Canada

**Keywords:** Healthcare use, Ambulatory surgery, Ontario, Administrative data, Cohort study

## Abstract

**Background:**

Advances in healthcare delivery have allowed for the increase in the number of ambulatory surgery procedures performed in Canada. Despite these advances, patients return to hospital following discharge. However, the reason for unplanned healthcare use after ambulatory surgery in Canada is not well understood.

**Aims:**

To examine unplanned healthcare use, specifically emergency department visit and hospital admissions, in the 3 days after ambulatory surgery in Ontario, Canada.

**Methods:**

This population-based retrospective cohort study was conducted using de-identified administrative databases. Participants were residents in the province of Ontario, Canada; 18 years and older; and underwent common ambulatory surgical procedures between 2014 and 2018. The outcomes included emergency department (ED) visit and hospital admission. Incidence rates were calculated for the total cohort, for each patient characteristic and for surgical category. The odds ratios and 95% confidence intervals were calculated for each outcome using bivariate and multivariate logistic regression.

**Results:**

484,670 adults underwent select common surgical procedures during the study period. Patients had healthcare use in the first 3 days after surgery, with 14,950 (3.1%) ED visits and 14,236 (2.9%) admissions. The incidence of ED use was highest after tonsillectomy (8.1%), cholecystectomy (4.2%) and appendectomy (4.0%). Incidence of admissions was highest after appendectomy (21%). Acute pain (19.7%) and haemorrhage (14.2%) were the most frequent reasons for an ED visit and “convalescence following surgery” (49.2%) followed by acute pain (6.2%) and haemorrhage (4.5%) were the main reasons for admission.

**Conclusions:**

These findings can assist clinicians in identifying and intervening with patients at risk of healthcare use after ambulatory surgery. Pain management strategies that can be tailored to the patient, and earlier follow-up for some patients may be required. In addition, administrative decision-makers could use the results to estimate the impact of specific ambulatory procedures on hospital resources for planning and allocation of resources.

## Background

Surgical procedures that previously required an in-patient post-surgical hospital stay are more frequently being performed as ambulatory surgery (planned for discharge on day of surgery). In Canada, from 1996 to 2007, the number of ambulatory surgery procedures increased by 31%, with over 1.3 million ambulatory surgery visits in 1995–1996 compared to approximately 1.8 million visits in 2005–2006 (Canadian Institute for Health Information, 2007). The rate of emergency department (ED) use has also increased over the past decade. In Ontario, from 2008 to 2015, the number of ED visits increased by 13.1%, while the population increased by only 6.2% (Health Quality Ontario, 2016). ED use and admissions after hospital discharge are considered a priority outcome in the Health Quality Ontario Quality Improvement Plan and more attention is being focused on strategies to reduce ED visits (Health Quality Ontario, 2015). One of the contributing factors to increased ED use may be related to increasing rates of ambulatory surgery.

In Canada and the United States (US), the rate of return to ED or hospital admission between 24 h and 14 days after ambulatory surgery ranges from 3.1 to 6.5% (Fox et al., 2014; McIsaac et al., 2015; Menedez & Ring, 2016). The rate of return to hospital within 30 days after surgery ranges from 3 to 10.5% and varies by surgical procedure (Bhattacharyya, 2014; Perron-Burdick et al., 2011; Steiner et al., 2015). Unrelieved pain and bleeding are the most common reasons for return to hospital (Fox et al., 2014; Menedez & Ring, 2016; Perron-Burdick et al., 2011; Steiner et al., 2015). Patients who are older than 50 years return to the ED more often than younger patients (Fox et al., 2014; Bhattacharyya, 2014; Perron-Burdick et al., 2011). The day of the week the patient undergoes surgery may also be a factor, with a Canadian study reporting that surgery performed on a Friday increased the risk (adjusted hazard ratio 1.07; 95% confidence interval (CI), 1.03–1.11) of return to hospital within 30 days after ambulatory surgery in patients older than 40 years (Bhattacharyya, 2014). Patients also report a lack of clear care instructions and a lack of timely follow-up after discharge from ambulatory surgery, which may contribute to healthcare use (Beauregard et al., 1998; Mattila et al., 2005; McHugh & Thoms, 2002; Oberle et al., 1994; Watt-Watson et al., 2004).

This literature provides some information regarding the utilization of emergency care services following ambulatory surgery. However, it is unclear how many patients utilize emergency room services in the first 3 days following discharge after ambulatory surgery, what the impact is to the healthcare system, and what the common reasons why patients return to hospital are.

## Methods

### Aim

The purpose of this study was to examine healthcare use (ED and admissions) in the first 3 days after ambulatory surgery in Ontario. Three days following surgery was chosen in an attempt to capture healthcare use most likely to be associated with surgery rather than other factors. The aims of this study were the following:

1. Calculate the incidence of healthcare use (ED and admissions) after common ambulatory surgery procedures
2. Identify patient characteristics and surgical groups associated with higher risk of healthcare use
3. Describe the main reasons for healthcare use overall and by surgical group

### Study design and participants

This population-based retrospective cohort study was designed according to the STROBE guidelines. It was conducted using de-identified administrative databases held by the Institute of Clinical Evaluative Sciences (IC/ES). Ontario-specific databases utilized included the Registered Persons Database (RPDB), Ontario Health Insurance Plan (OHIP), Ontario Marginalization Index (ON-MARG), Client Agency Program Enrolment database (CAPE) and Corporate Provider database (CPDB). The Canadian databases utilized included the Canadian Census, Canadian Institute for Health Information Same Day Surgery (CIHI-SDS), Discharge Abstract Database (CIHI-DAD) and National Ambulatory Care Reporting System (CIHI-NACRS) databases. These datasets were linked deterministically using unique encoded identifiers and were analysed at IC/ES. This study was approved by the institutional review board at Sunnybrook Health Sciences Centre, Toronto, Canada, and the Queen’s University Health Science and Affiliated Teaching Hospitals Research Ethics Board, Kingston, Canada.

Participants included adults aged 18 years and older residing in Ontario who underwent select ambulatory surgical procedures between April 1, 2014, and March 31, 2018. The selection of surgical procedures was adapted from the CIHI’s report of the most common ambulatory surgery procedures (Canadian Institute for Health Information, 2012). Included surgical procedures were hernia-related muscle repair of the chest and abdomen, cholecystectomy, knee joint repair, release of nerves in the forearm, shoulder surgery, tonsillectomy, tympanic membrane procedures, appendectomy and partial hysterectomy. Description of the specific diagnostic and surgical procedures that fall under these surgical categories is included in Appendix A. Patients were excluded if they had an ED visit immediately prior to their surgery to ensure that only elective surgical procedures were included. They were also excluded if they did not have a valid OHIP number or if they did not have Ontario health insurance coverage in the year preceding surgery. The latter was required to allow for calculation of pre-operative comorbidities. Patients who died on the day of surgery were also excluded. If patients underwent more than one ambulatory surgery between 2014 and 2018 only their first ambulatory surgery was included as prior ambulatory surgery experience could influence post-operative self-care and experience.

The outcomes of interest were healthcare use (unplanned ED visits and admissions) in the first 3 days following ambulatory surgery and the main reason for ED visit or hospital admission. This information was captured from NACRS or DAD. Hospital admission following surgery was confirmed by cross-referencing between SDS database (confirmed procedure booked as ambulatory) and the DAD (confirmed time of admission to hospital following surgery).

The main exposure variable, type of surgical procedure, was captured from the SDS Database. The Canadian Classification of Health Interventions (CCI) codes were used to classify surgical procedures (Canadian Institute for Health Information, n.d.). The CCI codes and companion surgical procedures are provided in Appendix A.

Demographic characteristics included age, sex, rurality of residence based on Rurality Index of Ontario 2008 and Local Health Integration Network (LHIN) (Local Health Integration Network, 2014). All were captured from the RPDB and the 2006 Canadian Census. Individual level measures of socioeconomic status were not available in the databases. Therefore, material deprivation was captured from the ON-MARG database. The ON-MARG database provides aggregate level measures of socioeconomic status based on the neighbourhood and considers variation in education, income and family composition (Durbin et al., 2015). The model of the usual provider of primary care (Government of Ontario, 2017; Glazier et al., 2015) was taken from CAPE and CPDB databases. Comorbidity in the year prior to surgery was measured using the Johns Hopkins Aggregated Diagnostic Groups (ADGs). ADGs were captured from DAD, NACRS and OHIP and were classified as major or minor (Johns Hopkins University ACG System, Version 10) (The Johns Hopkins University, 2017).

### Data analysis

Demographic and clinical characteristics were summarized using measures of central tendency and spread, or frequencies and percentages, as appropriate. Listwise deletion was utilized, given the low frequency of missing data. The incidence of at least one ED visit or hospital admission was calculated for the total cohort and according to patient characteristics and surgical category. Bivariate and multivariate logistic regression analyses were used to calculate the odds ratios (OR) and 95% confidence intervals (CI) for ED use and hospital admission. In this study, odds ratios are used as a proxy of risk because incidence is rare (< 10%) (Viera, 2008). Cholecystectomy was selected as the reference surgery for the purpose of interpreting the odds ratios. The rationale for selecting cholecystectomy was that sample size was sufficient for meaningful comparisons with other surgical procedures. The full adjusted models included all available variables; age, sex, primary care model, LHIN, material deprivation quintile, rural/urban residence, comorbidity (major ADGs) and surgical category. The main reasons for ED use were calculated for all surgical procedures combined and for those surgical procedures with sufficient sample size and volume to avoid small cell frequencies. Hospital admissions were only calculated for all surgical procedures combined due to the small cell frequencies. All analyses were conducted using SAS© (SAS Enterprise Guide, Version 7.1).

### Results

**Fig. 1:**
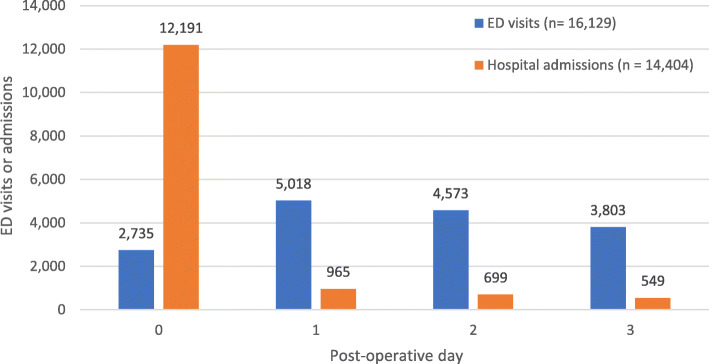
Distribution of emergency department visits and hospital admission by post-operative day: 2014–2018. All of the emergency department (ED) visits and hospital admissions are displayed as proportions based on the post-operative day. The majority of ED visits were made on post-operative day 1 (5018; 33.6%) or 2 (4573; 30.6%). The majority of hospital admissions occurred on the same day of surgery (post-operative day 0) (*n* = 12,191, 85.6%)

Over four hundred and eighty thousand (484,670) adults underwent the selected surgical procedures in Ontario between April 1, 2014, and March 31, 2018. The mean age of study participants was 51.80 ± 15.46 years, 56.6% were female and 20.6% had at least 2 major comorbidities (ADGs; Table 1). The most frequently performed surgical procedures were hernia repair (22.9%) and partial hysterectomy (21.1%). A total of 29,186 (6.0%) patients utilized healthcare through an ED visit or hospital admission in the first 3 days after surgery, with 14,950 (3.1%) making an ED visit and 14,236 (2.9%) admitted to hospital (through the ED or directly). Approximately eight percent (7.9%, *n* = 1179/14,950) of patients who visited the ED returned more than once. The majority of ED visits were made on post-operative day 1 (5018; 33.6%) or 2 (4573; 30.6%) (Fig. 1). Of the patients admitted to hospital 1.2% (168/14,404) were admitted more than once in the 3 days following surgery. The majority of patients who were admitted to hospital were scheduled for an ambulatory surgery procedure with same day discharge; however, they were not discharged as planned after their scheduled ambulatory surgery (*n* = 12,191, 85.6%).

**Table 1:** Demographic and clinical characteristics of adults who underwent ambulatory surgery in Ontario between 2014 and 2018

Characteristics	Total, *N* = 484,670	ED visits, *N* = 14,950	Hospital admission, *N* = 14,236
	*n* (%)	*n* (%)	*n* (%)
Mean age (± SD)	51.80 (± 15.46)	50.74 (± 18.04)	56.07 (± 17.08)
Age categories			
18–40	116,114 (24)	4680 (31.3)	2853 (20.1)
41–60	225,152 (46.5)	5397 (36.1)	5186 (36.4)
61–70	86,486 (17.8)	2543 (17.0)	3079 (21.6)
71–90+	56,918 (11.7)	2330 (15.6)	3118 (21.9)
Sex			
Female	274,247 (56.6)	7414 (49.6)	7762 (54.5)
Male	210,423 (43.4)	7536 (50.4)	6474 (45.5)
Material deprivation quintile			
1—lowest	104,530 (21.6)	2699 (18.1)	2555 (17.9)
2	102,621 (21.2)	2920 (19.5)	2795 (19.6)
3	95,956 (19.8)	2866 (19.2)	2795 (19.6)
4	90,662 (18.7)	2973 (19.9)	2889 (20.3)
5—highest	87,529 (18.1)	3270 (21.9)	3005 (21.1)
Missing	3372 (0.7)	222 (1.5)	197 (1.4)
Residence^*^			
Urban	416,877 (86.0)	12,069 (80.7)	11,622 (81.6)
Rural	67,237 (13.9)	2867 (19.2)	2597 (18.2)
Missing	556 (0.1%)	14 (0.1%)	17 (0.1%)
Number of major ADGs			
0	207,310 (42.8)	5389 (36.0)	4354 (30.6)
1	177,491 (36.6)	5307 (35.5)	4743 (33.3)
2+	99,869 (20.6)	4254 (28.5)	5139 (36.1)
Usual provider of care model			
Family Health Group^†^	115,522 (23.8)	3265 (21.8)	3065 (21.5)
Family Health Team^‡^	142,311 (29.4)	4778 (32.0)	4457 (31.3)
Family Health Organization^II^	141,696 (29.2)	3954 (26.4)	3851 (27.1)
No model	66,391 (13.7)	2364 (15.8)	2216 (15.6)
Comprehensive Care Model^**^	14,184 (2.9)	402 (2.7)	466 (3.3)
Other	4551 (0.9)	186 (1.2)	181 (1.3)
Type of surgery			
Muscle repair of the chest and abdomen: hernia	110,802 (22.9)	4441 (29.7)	3875 (27.2)
Partial hysterectomy	102,073 (21.1)	1222 (8.2)	1443 (10.1)
Cholecystectomy	82,891 (17.1)	3463 (23.2)	5417 (38.1)
Knee joint repair	81,000 (16.7)	1702 (11.4)	509 (3.6)
Nerves in the forearm and wrist	40,340 (8.3)	1090 (7.3)	248 (1.7)
Shoulder surgery	41,537 (8.6)	1444 (9.7)	1187 (8.3)
Appendectomy	4444 (0.9)	179 (1.2)	950 (6.7)
Implantation of internal devices, tympanic membrane	5156 (1.1)	81 (0.5)	30 (0.2)
Tonsillectomy	16,427 (3.4)	1328 (8.9)	577 (4.1)

*Estimates based on Rurality Index of Ontario 2008

^†^Family Health Groups are groups of 3 or more family MDs. Care is provided through regular office hours and extended hours (weekday evenings and/or weekends) and they utilize fee-for-service plus some incentives and bonuses for services provided to enrolled patients (Government of Ontario, 2017)

^‡^Family Health Teams are community-focused primary healthcare organizations that consist of interprofessional teams including MDs, nurse practitioners, registered nurses, social workers, dietitians and other professionals who work together. Physicians are paid through a blended salary model. Other health professionals are paid through salary (Government of Ontario, 2017)

^II^Family Health Organizations are groups of 3 or more family MDs who commit to enrol patients; care provided through regular office hours and extended hours based on the number of physicians; services are paid through a blended capitation model plus some incentives and bonuses for services to enrolled patients

^**^Comprehensive Care Models are solo primary care MD’s; care is provided through regular office hours plus at least one session of extended hours weekly and utilize fee-for-service plus some incentives and bonuses for service (Government of Ontario, 2017)

**Fig. 2:**
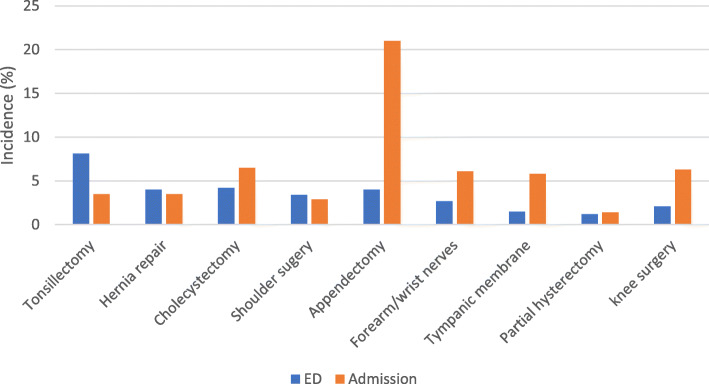
Incidence of emergency department visits and hospital admissions post-operatively by type of surgery: 2014–2018. Emergency department visits and hospital admissions by type of surgery are displayed in proportions. All ED visits and hospital admissions from day of surgery (post-operative day 0) to post-operative day 3 are included. Patients undergoing tonsillectomy had the highest incidence of ED use (8.1%), followed by cholecystectomy (4.2%), hernia repair (4.0%) or appendectomy (4.0%). The highest incidence of admission to hospital was for patients who underwent appendectomy (21%)

Of the 14,950 patients who visited the ED at least once, the majority underwent hernia repair (29.7%) or cholecystectomy (24.6%) (Table 1). Of the 14,236 patients who were admitted to hospital at least once, the majority underwent cholecystectomy (38.1%) or hernia repair (27.2%). The high percentage of visits in these two surgical groups can be attributed to the fact that they made up 40% of surgical procedures conducted during the study period. The incidence of patients who had at least one ED visit or hospital admission according to surgery type is presented in Fig. 2. Patients undergoing tonsillectomy had the highest incidence of ED use (8.1%), followed by cholecystectomy (4.2%), hernia repair (4.0%) or appendectomy (4.0%). The highest incidence of admission to hospital was for patients who underwent appendectomy (21%).

Females had a lower odds of ED use compared to males (adjusted OR = 0.73, CI 0.71–0.76) (Table 2). Patients had a higher odds of ED use if they lived in a rural setting (adjusted OR = 1.49, CI 1.43–1.56) or had a poor socioeconomic status (adjusted OR = 1.38, CI 1.31–1.46; reference group was lowest level of material deprivation). The odds of ED use also increased as number of major comorbidities increased (2+ ADGs OR = 1.78, CI 1.70–1.85). Patients who underwent tonsillectomy had the highest odds of ED use (OR 1.66, CI 1.55–1.77, reference = cholecystectomy).

**Table 2:** Univariate and multivariate odds ratios and 95% confidence intervals for ED visit and hospital admissions in the 3 days following ambulatory surgery: 2014–2018

Character	Total	ED Visits		Hospital Admissions	
		Unadjusted	Adjusted^‡^	Unadjusted	Adjusted^‡^
		OR (95% CI)	OR (95% CI)	OR (95% CI)	OR (95% CI)
Number of patients	484,670				
Sex					
Male	210,423	1.00	1.00	1.00	1.00
Female	274,247	0.75 (0.72–0.77)	0.73 (0.71–0.76)	0.92 (0.89–0.95)	1.00 (0.97–1.04)
Material deprivation quintile					
1—lowest (reference)	104,530	1.00	1.00	1.00	1.00
2	102,621	1.10 (1.05–1.17)	1.08 (1.03–1.14)	1.12 (1.06–1.18)	1.11 (1.05–1.18)
3	95,956	1.16 (1.10–1.23)	1.13 (1.07–1.19)	1.20 (1.13–1.26)	1.18 (1.12–1.25)
4	90,662	1.28 (1.21–1.35)	1.22 (1.15–1.29)	1.31 (1.24–1.39)	1.29 (1.22–1.36)
5—highest	87,529	1.46 (1.39–1.54)	1.38 (1.31–1.46)	1.42 (1.34–1.50)	1.44 (1.36–1.52)
Missing	3372	2.66 (2.31–3.07)	1.99 (1.72–2.30)	2.48 (2.13–2.88)	2.45 (2.10–2.85)
Residence^*^					
Urban (reference)	416,877	1.00	1.00	1.00	1.00
Rural	67,237	1.49 (1.43–1.56)	1.28 (1.22–1.34)	1.40 (1.34–1.46)	1.25 (1.19–1.31)
# of Major ADG^†^					
0 (reference)	207,310	1.00	1.00	1.00	1.00
1	177,491	1.15 (1.11–1.20)	1.19 (1.14–1.23)	1.28 (1.23–1.33)	1.24 (1.19–1.30)
2	99,869	1.67 (1.60–1.74)	1.78 (1.70–1.85)	2.53 (2.43–2.64)	2.28 (2.19–2.38)
Usual provider of care model					
No model (reference)	66,391	1.00	1.00	1.00	1.00
Family Health Group	115,522	0.79 (0.75–0.83)	0.87 (0.82–0.92)	0.79 (0.75–0.83)	0.79 (0.75–0.84)
Family Health Team	142,311	0.94 (0.89–0.99)	0.91 (0.87–0.96)	0.94 (0.89–0.99)	0.85 (0.81–0.90)
Family Health Organization	141,696	0.78 (0.74–0.82)	0.82 (0.78–0.87)	0.81 (0.77–0.85)	0.76 (0.72–0.80)
Comprehensive Care Model	14,184	0.79 (0.71–0.88)	0.84 (0.75–0.93)	0.98 (0.89–1.09)	0.94 (0.85–1.04)
Other	4551	1.15 (0.99–1.34)	0.87 (0.74–1.02)	1.20 (1.03–1.40)	0.97 (0.83–1.14)
Type of surgery					
Cholecystectomy (reference)	82,891	1.00	1.00	1.00	1.00
Implantation of internal devices, tympanic membrane	5156	0.37 (0.29–0.46)	0.36 (0.29–0.45)	0.08 (0.06–0.12)	0.08 (0.05–0.11)
Knee joint repair	81,000	0.49 (0.46–0.52)	0.50 (0.47–0.53)	0.09 (0.08–0.10)	0.09 (0.08–0.10)
Nerves in the forearm and wrist	40,340	0.64 (0.59–0.68)	0.60 (0.56–0.65)	0.09 (0.08–0.10)	0.07 (0.06–0.08)
Shoulder surgery	41,537	0.83 (0.78–0.88)	0.83 (0.78–0.88)	0.42 (0.39–0.45)	0.39 (0.37–0.42)
Muscle repair of the chest/abdomen: hernia	110,802	0.96 (0.92–1.00)	0.97 (0.93–1.02)	0.52 (0.50–0.54)	0.44 (0.42–0.46)
Appendectomy	4444	0.96 (0.83–1.12)	0.94 (0.81–1.10)	3.89 (3.60–4.20)	4.64 (4.28–5.02)
Tonsillectomy	16,427	2.02 (1.89–2.15)	1.95 (1.82–2.09)	0.52 (0.48–0.57)	0.91 (0.83–1.00)

*Estimates based on Rurality Index of Ontario 2008

^†^Johns Hopkins Aggregated Diagnostic Groups

^‡^Adjusted for age and Local Health Integration Network (results not shown)

Acute pain (including abdominal pain; 19.7%) and haemorrhage (14.2%) were the most frequent reasons for an ED visit (Table 3). Table 4 provides a more detailed breakdown of the 5 most frequent reasons for an ED visit for the 7 surgical procedures with the highest incidence of visits. The primary reason for admission to hospital was coded by providers as “convalescence following surgery” (49.2%), followed by acute pain (6.2%) and haemorrhage/hematoma (4.5%). Due to small cell frequencies, the results for hospital admission for specific surgical procedures are not presented.

**Table 3:** Ten most common reasons for emergency department visit and hospital admission for all procedures combined: 2014–2018

Reasons for ED visit (n=14,950)	n	% of visits
Acute pain	2711	16.8
Haemorrhage and haematoma	2287	14.2
Retention of urine	1119	6.9
Other complications of procedures, not elsewhere classified	819	5.1
Attention to surgical dressings and sutures	813	5.0
Constipation	492	3.1
Other and unspecified abdominal pain	463	2.9
Infection following a procedure	436	2.7
Follow-up examination after surgery for other conditions	347	2.2
Vomiting	236	1.5
Reasons for hospital admission (n=14,236)	n	% of admissions
Convalescence following surgery	7086	49.2
Acute pain	893	6.2
Haemorrhage and haematoma	655	4.5
Accidental puncture and laceration during a procedure	267	1.9
Retention of urine	265	1.8
Abnormal findings of blood chemistry	251	1.7
Postoperative intestinal obstruction	224	1.6
Infection following a procedure	186	1.3
Other complications of procedures, not elsewhere classified	171	1.2
Postoperative leak	160	1.1

**Table 4:** Top 5 reasons for ED visits for surgical procedures with highest rate of hospital use

Procedure (number of visits)	*n*	% of ED visits/procedure
Hernia	4831	
Haemorrhage and haematoma	888	18.4
Retention of urine	569	11.8
Acute pain	505	10.5
Other complications of procedures, not elsewhere classified	290	6.0
Constipation	269	5.6
Cholecystectomy	3772	
Acute pain	715	19.0
Haemorrhage and haematoma	413	10.9
Retention of urine	341	9.0
Other and unspecified abdominal pain*	209	5.5
Attention to surgical dressings and sutures	185	4.9
Knee repair	1826	
Haemorrhage and haematoma	427	23.4
Acute pain	263	14.4
Other complications of procedures, not elsewhere classified	82	4.5
Attention to surgical dressings and sutures	81	4.1
Phlebitis and thrombophlebitis of other deep vessels of lower extremity	74	4.1
Shoulder surgery	1566	
Acute pain	497	31.7
Retention of urine	110	7.0
Attention to surgical dressings and sutures	89	5.7
Haemorrhage, haematoma	77	4.9
Other complications of procedures, not elsewhere classified	60	3.8
Tonsillectomy	1412	
Acute pain	374	26.5
Haemorrhage, haematoma	293	20.8
Other complications of procedures, not elsewhere classified	117	8.3
Vomiting alone	90	6.4
Nausea and vomiting	56	4.0
Partial Hysterectomy	1299	
Acute pain	141	10.9
Haemorrhage and haematoma	111	8.5
Other and unspecified abdominal pain*	70	5.4
Urinary tract infection	50	3.8
Retention of urine	49	3.8
Nerves in forearm and wrist	1147	
Attention to surgical dressings and sutures	197	17.2
Acute pain	159	13.9
Follow-up examination after surgery for other conditions	69	6.0
Other complications of procedures, not elsewhere classified	65	5.7
Haemorrhage and haematoma	58	5.1

*Other pain-related visits was calculated by summing all reasons (except acute pain) with a pain-related component

## Discussion

The findings of this retrospective cohort study shed light on the rate of healthcare use after ambulatory surgery in Ontario. Between 2014 and 2018, 3.1% of patients visited the ED and 2.9% were admitted to hospital during the first 3 days following select ambulatory procedures. Due to high surgical volume, patients who underwent hernia repair or cholecystectomy had the highest number of healthcare visits. However, the highest incidence of use was in patients who underwent tonsillectomy (ED) and appendectomy (admission). The main reason for ED use for all surgery types was unrelieved acute pain or bleeding, while for hospital admissions it was convalescence (with no defined reason for admission), followed by acute pain and bleeding. A large number of patients were scheduled for same day discharge, however they were not discharged as planned after their scheduled ambulatory surgery. These unplanned hospital admissions have an impact on hospital resources, including bed allocation and staffing.

### Explanation of the findings

Our findings are consistent with studies in the US where 3.1% of patients accessed hospital care within the first 7 days following their ambulatory medical or surgical procedure (Fox et al., 2014). The most common reasons for accessing hospital care following discharge were also consistent with our findings; complications of the operative procedure (i.e. bleeding) or pain or discomfort. However, in the US study, the majority of patients had an ED visit on the day of surgery (Fox et al., 2014), and we found that an ED visit occurred most often on post-operative day 1 (33.1%) and less frequently on the day of surgery (18.3%).

The findings from our study are also similar to US studies that examine healthcare use after ambulatory tonsillectomy and hysterectomy. Bhattacharyya’s findings that 5.5% of adult patients who had tonsillectomy as an ambulatory surgery had a revisit after surgery (i.e. ambulatory surgery centre, ED or hospital admission) is similar to our study where 4.1% of our sample who underwent tonsillectomy were admitted and 8.9% had an ED visit (Bhattacharyya, 2014). The primary diagnoses at the first revisit were also consistent with our findings, acute pain (26.5%) and bleeding (20.8%). Perron-Burdick et al. examined readmission rates and emergency care use after ambulatory laparoscopic hysterectomy in California from 2007 to 2009 and reported 4% ED use within 72 h following discharge (Perron-Burdick et al., 2011). The most common reasons for emergency care were urinary retention, pain, nausea and vomiting. The ED visit rate for partial hysterectomy was higher in our study (10.9%) compared to Perron-Burdick et al. (Perron-Burdick et al., 2011) (0.6% at 48 h), but pain and urinary retention were the main reason for ED use.

CIHI reported that post-surgical related issues are one of the 10 most common reasons for ED visits for people between 18 to 85 years old or older accounting for 5 to 11% of potentially avoidable ED visits (Canadian Institute of Health Information, 2014). Not all ED visits and hospital admissions can be avoided; however, it has not been determined what the clinically acceptable rate of ED visits or hospital admissions is after ambulatory surgery. ED’s are designed to prioritize care for patients who are critically ill or who have emergency needs who require timely, highly skilled care (Canadian Institute of Health Information, 2014). In addition, ED’s are challenged to do more with less resources, including space, staff and equipment (Beveridge et al., 1998). Unfortunately, reasons for healthcare use for Ontario patients after ambulatory surgery have not changed in the past 16 years. Two studies conducted in the early 2000’s (2002 and 2004) in Ontario, Canada, reported that after discharge from hospital patients contacted a nurse or doctor or had an ED visit due to pain (including asking for information on how to take analgesics), bleeding, or nausea and vomiting (Oberle et al., 1994; McGrath et al., 2004). Pain continues to be the most common reason for ED visits after ambulatory surgery despite the availability of guidelines and resources for clinicians (Health Quality Ontario, 2018; Chou et al., 2016).

### Strengths and limitations

Strengths of this study include the use of administrative data allowing for examination of population level characteristics with minimal missing information; therefore, the results are representative of patients undergoing the selected surgical procedures on an ambulatory basis in the province of Ontario. Databases used in this study undergo quality checks by several data collection and repository organizations, such as CIHI and IC/ES thereby providing a level of reliability. The results could be generalized with caution to the rest of Canada, where universal healthcare provides similar access to services. The ability to link several administrative databases provided an opportunity to adjust for patient characteristics when examining healthcare use after ambulatory surgery. The use of administrative data is also a strength because it does not rely on patient reports of past experiences and decreases the risk of recall bias.

The disadvantage of administrative data is that it relies on accurate coding and recording of information and it lacks clinical detail. This affected the ability to identify the primary reason for hospital admission. Forty-eight percent of admissions were coded with the very general term “convalescence after surgery”. Based on discussions with clinicians, this term includes many admission reasons ranging from the patient requiring post-operative monitoring due to unstable vital signs or multiple comorbidities, to the patient requiring an overnight stay due to patient safety concerns related to living alone. Further analysis of individual patient records is needed to explore the true meaning of “convalescence after surgery” as we were unable to determine this utilizing administrative data. In addition, post-operative pain was coded inconsistently; in some cases, it was recorded acute pain and other times it was reported by location (e.g. “pain in lower limb” in someone who underwent knee surgery) potentially resulting in the underreporting of pain. Earlier studies examining pain following ambulatory surgery report that 21 to 62% of patients have moderate to severe pain on the day of surgery and 18 to 44% report moderate to severe pain 24 h after surgery (Oberle et al., 1994; McGrath et al., 2004). We were unable to obtain this level of detail and analysis of the individual patient record would also be required to obtain a better understanding of the severity of pain.

## Conclusion and future directions

The results of this study have implications for knowledge users at several levels. Clinicians may use the results to identify patients at high risk of ED use or hospital admission and implement targeted interventions on high-risk groups. For example, evidenced-based pain management strategies that can be tailored to the patient’s needs should be considered.

Pain management strategies may include patient education, the use of local anaesthetics pre-operatively and intra-operatively, multimodal analgesia and non-pharmacological pain management strategies (Health Quality Ontario, 2018; Chou et al., 2016). Also, a re-evaluation of the timing and method of follow-up visits could be reassessed, especially for patients who are older or have multiple comorbidities. Administrative decision-makers could use the results to estimate the impact of specific ambulatory procedures on hospital resources for better planning and allocation of the health workforce. Administrators can re-evaluate the appropriateness of classifying procedures such as appendectomy as ambulatory surgery (with same day surgery and discharge). Future studies are needed to clarify the primary reasons for healthcare use, to estimate the cost of healthcare use after ambulatory surgery and to assess the impact and efficiency of implementing interventions to reduce use, such as pre-operative educational interventions.

## Supplementary information

**Additional file 1.** Canadian Classification of Health Interventions codes for day surgery procedures
