# Supplementary material for: Pain and haemorrhage are the most common reasons for emergency department use and hospital admission in adults following ambulatory surgery: results of a population-based cohort study
Source: Perioper Med (Lond). 2020 Aug 19;9:25. doi: 10.1186/s13741-020-00155-3 (PMC7436986; doi:10.1186/s13741-020-00155-3)
Supplement: Supplementary file 1 — Additional file 1. Canadian Classification of Health Interventions codes for day surgery procedures [file 13741_2020_155_MOESM1_ESM.zip › Additional file 1.docx]

Additional file 1: Canadian Classification of Health Interventions codes for day surgery procedures

| **Code** | **Description** |
| --- | --- |
| **Muscle repair of the chest and abdomen** | |
| 1SY80DA | Repair, muscles of the chest and abdomen endoscopic [laparoscopic] approach without tissue [e.g. suturing or stapling] |
| 1SY80DAXXA | Repair, muscles of the chest and abdomen endoscopic [laparoscopic] approach using autograft [e.g. fascia, skin] |
| 1SY80DAXXF | Repair, muscles of the chest and abdomen endoscopic [laparoscopic] approach using free flap [e.g. free myocutaneous flap] |
| 1SY80DAXXG | Repair, muscles of the chest and abdomen endoscopic [laparoscopic] approach using pedicled flap [e.g. abdominis rectus or deltopectoral] |
| 1SY80DAXXL | Repair, muscles of the chest and abdomen, endoscopic [laparoscopic] approach using xenograft [e.g. Surgis, SIS (small intestine submucosa)] |
| 1SY80DAXXN | Repair, muscles of the chest and abdomen endoscopic [laparoscopic] approach using synthetic tissue [e.g. mesh, sponge] |
| 1SY80LA | Repair, muscles of the chest and abdomen open approach without tissue [e.g. suturing or stapling] |
| 1SY80LAFF | Repair, muscles of the chest and abdomen open approach and using temporary abdominal closure device [e.g. Bogota bag] |
| 1SY80LATZ | Repair, muscles of the chest and abdomen open approach using zipper [temporary] (for repeat access to abdomen) |
| 1SY80LAXXA | Repair, muscles of the chest and abdomen open approach using autograft [e.g. fascia, skin] |
| 1SY80LAXXF | Repair, muscles of the chest and abdomen open approach using free flap [e.g. free myocutaneous flap] |
| 1SY80LAXXG | Repair, muscles of the chest and abdomen open approach using pedicled flap [e.g. abdominis rectus or deltopectoral] |
| 1SY80LAXXL | Repair, muscles of the chest and abdomen, open approach using xenograft [e.g. Surgisis, SIS (small intestine submucosa)] |
| 1SY80LAXXN | Repair, muscles of the chest and abdomen open approach using synthetic tissue [e.g. mesh, sponge] |
| 1SY80LAXXQ | Repair, muscles of the chest and abdomen open approach and combined sources of tissue (e.g. mesh with autograft) |
| 1SY80PN | Repair, muscles of the chest and abdomen robotic assisted telemanipulation of tools [telesurgery] without tissue [e.g. suturing or stapling] |
| 1SY80PNXXN | Repair, muscles of the chest and abdomen robotic assisted telemanipulation of tools [telesurgery] using synthetic tissue [e.g. mesh, sponge] |
| 1SY80WJ | Repair, muscles of the chest and abdomen open approach using special excisional technique |
| **Partial hysterectomy** | |
| 1RM87BAAG | Excision partial, uterus and surrounding structures endoscopic per orifice [hysteroscopic] approach using laser |
| 1RM87BAAK | Excision partial, uterus and surrounding structures endoscopic per orifice [hysteroscopic] approach using loop electrode [LEEP] |
| 1RM87BAGX | Excision partial, uterus and surrounding structures endoscopic per orifice [hysteroscopic] approach using device NEC |
| 1RM87CAAE | Excision partial, uterus and surrounding structures per orifice [transvaginal] approach using curette (e.g. D & C) |
| 1RM87CAAF | Excision partial, uterus and surrounding structures per orifice [transvaginal] approach using aspiration curettage |
| 1RM87CAAK | Excision partial, uterus and surrounding structures per orifice [transvaginal] approach using loop electrode [LEEP] |
| 1RM87CAGX | Excision partial, uterus and surrounding structures per orifice [transvaginal] approach using device NEC |
| 1RM87DAAG | Excision partial, uterus and surrounding structures endoscopic [laparoscopic] approach using laser |
| 1RM87DAAK | Excision partial, uterus and surrounding structures endoscopic [laparoscopic] approach using loop electrode [LEEP] |
| 1RM87DAGX | Excision partial, uterus and surrounding structures endoscopic [laparoscopic] approach using device NEC |
| 1RM87LAAK | Excision partial, uterus and surrounding structures open approach using loop electrode [LEEP] |
| 1RM87LAGX | Excision partial, uterus and surrounding structures open approach using device NEC |
| **Cholecystectomy** | |
| 1OD89DA | Excision total, gallbladder endoscopic [laparoscopic] approach without extraction (of calculi) cholecystectomy alone |
| 1OD89DTAG | Excision total, gallbladder endoscopic [laparoscopic] approach with extraction (of calculi) from bile ducts using laser probe |
| 1OD89DTAM | Excision total, gallbladder endoscopic [laparoscopic] approach with extraction (of calculi) from bile ducts using basket device |
| 1OD89DTAS | Excision total, gallbladder endoscopic [laparoscopic] approach with extraction (of calculi) from bile ducts using electrohydraulic probe |
| 1OD89DTBD | Excision total, gallbladder endoscopic [laparoscopic] approach with extraction (of calculi) from bile ducts using balloon device |
| 1OD89DTGX | Excision total, gallbladder endoscopic [laparoscopic] approach with extraction (of calculi) from bile ducts using device NEC [e.g. forceps, metal probe] |
| 1OD89EC | Excision total, gallbladder endoscopic [laparoscopic] approach cholecystectomy with bile duct exploration and no stones extracted |
| 1OD89LA | Excision total, gallbladder open approach without extraction of calculi cholecystectomy alone |
| 1OD89PN | Excision total, gallbladder robotic assisted telemanipulation of tools [telesurgery] without extraction of calculi cholecystectomy alone |
| 1OD89SMAG | Excision total, gallbladder open approach with extraction (of calculi) from bile ducts using laser probe |
| 1OD89SMAM | Excision total, gallbladder open approach uwith extraction (of calculi) from bile ducts sing basket device |
| 1OD89SMAS | Excision total, gallbladder open approach with extraction (of calculi) from bile ducts using electrohydraulic probe |
| 1OD89SMBD | Excision total, gallbladder open approach with extraction (of calculi) from bile ducts using balloon device |
| 1OD89SMGX | Excision total, gallbladder open approach with extraction (of calculi) from bile ducts using device NEC [e.g. forceps, metal probe] |
| 1OD89TP | Excision total, gallbladder open approach cholecystectomy with bile duct exploration and no stones extracted |
| **Repair, knee joints** | |
| 1VG80DA | Repair, knee joint endoscopic [arthroscopic] approach no tissue used (for repair) joint repair without meniscus involvement |
| 1VG80DAAG | Repair, knee joint using endoscopic approach and laser NEC |
| 1VG80DAXXA | Repair, knee joint endoscopic [arthroscopic] approach with autograft [e.g. bone, cartilage, or tendon] joint repair without meniscus involvement |
| 1VG80DAXXK | Repair, knee joint endoscopic [arthroscopic] approach with homograft graft [e.g. bone or cartilage] joint repair without meniscus involvement |
| 1VG80DAXXN | Repair, knee joint endoscopic [arthroscopic] approach with synthetic tissue [e.g. gortex, artificial polymer cartilage] joint repair without meniscus involvement |
| 1VG80DAXXQ | Repair, knee joint endoscopic [arthroscopic] approach with combined sources of tissue [e.g. bone graft, synthetic tissue] joint repair without meniscus involvement |
| 1VG80FY | Repair, knee joint endoscopic [arthroscopic] approach no tissue used (for repair) with meniscectomy [or meniscoplasty] |
| 1VG80FYXXA | Repair, knee joint endoscopic [arthroscopic] approach with autograft [e.g. bone, cartilage, or tendon] with meniscectomy [or meniscoplasty] |
| 1VG80FYXXK | Repair, knee joint endoscopic [arthroscopic] approach with homograft graft [e.g. bone or cartilage] with meniscectomy [or meniscoplasty] |
| 1VG80FYXXN | Repair, knee joint endoscopic [arthroscopic] approach with synthetic tissue [e.g. gortex, artificial polymer cartilage] with meniscectomy [or meniscoplasty] |
| 1VG80FYXXQ | Repair, knee joint endoscopic [arthroscopic] approach with combined sources of tissue [e.g. bone graft, synthetic tissue] with meniscectomy [or meniscoplasty] |
| 1VG80GZ | Repair, knee joint, endoscopic (arthroscopic) approach using special incisional technique [e.g. multiple burr holes for tibial head revascularization] |
| 1VG80GZXXK | Repair, knee joint using endoscopic (arthroscopic) approach with homograft [e.g. bone or cartilage] using special incisional technique [e.g. multiple burr holes for tibial head revascularization] |
| 1VG80LA | Repair, knee joint open approach no tissue used (for repair) joint repair without meniscus involvement |
| 1VG80LAXXA | Repair, knee joint open approach with autograft [e.g. bone, cartilage, or tendon] joint repair without meniscus involvement |
| 1VG80LAXXK | Repair, knee joint open approach with homograft graft [e.g. bone or cartilage] joint repair without meniscus involvement |
| 1VG80LAXXN | Repair, knee joint open approach with synthetic tissue [e.g. gortex, artificial polymer cartilage] joint repair without meniscus involvement |
| 1VG80LAXXQ | Repair, knee joint open approach with combined sources of tissue [e.g. bone graft, synthetic tissue] joint repair without meniscus involvement |
| 1VG80UY | Repair, knee joint open approach no tissue used (for repair) with meniscectomy [or meniscoplasty] |
| 1VG80UYXXA | Repair, knee joint open approach with autograft [e.g. bone, cartilage, or tendon] with meniscectomy [or meniscoplasty] |
| 1VG80UYXXK | Repair, knee joint open approach with homograft graft [e.g. bone or cartilage] with meniscectomy [or meniscoplasty] |
| 1VG80UYXXN | Repair, knee joint open approach with synthetic tissue [e.g. gortex, artificial polymer cartilage] with meniscectomy [or meniscoplasty] |
| 1VG80UYXXQ | Repair, knee joint open approach with combined sources of tissue [e.g. bone graft, synthetic tissue] with meniscectomy [or meniscoplasty] |
| 1VG80WK | Repair, knee joint, open approach using special incisional technique [e.g. multiple burr holes for tibial head revascularization] |
| 1VG80WKXXK | Repair, knee joint using open approach with homograft [e.g. bone or cartilage] using special incisional technique [e.g. multiple burr holes for tibial head revascularization] |
| **Implantation of Internal Devices, tympanic membrane** | |
| 1DF53JATS | Implantation of internal device, tympanic membrane of ventilation [grommet] tube using external approach |
| **Nerves in the forearm and wrist** | |
| 1BN72DA | Release, nerve(s) of forearm and wrist using endoscopic approach |
| 1BN72LA | Release, nerve(s) of forearm and wrist using open approach |
| 1BN80LA | Repair, nerve(s) of forearm and wrist using end to end suture [rejoining] technique |
| 1BN80LAW3 | Repair, nerve(s) of forearm and wrist using fibrin glue [rejoining] technique |
| 1BN80UH | Repair, nerve(s) of forearm and wrist using interfascicular [split] repair [rejoining] technique |
| 1BN87LA | Excision partial, nerve(s) of forearm and wrist end to end [rejoining] technique (e.g. suture, glue) simple apposition of nerve ends |
| 1BN87LAXXA | Excision partial, nerve(s) of forearm and wrist end to end [rejoining] technique (e.g. suture, glue) nerve autograft (to replace lost length) |
| 1BN87LAXXE | Excision partial, nerve(s) of forearm and wrist end to end [rejoining] technique (e.g. suture, glue) transposition of nerves [e.g. crossover] |
| 1BN87LAXXN | Excision partial, nerve(s) of forearm and wrist, no rejoining [of nerve ends] nerve end(s) wrapped or bridged using synthetic tissue [e.g. neural tube] |
| 1BN87LAXXQ | Excision partial, nerve(s) of forearm and wrist end to end [rejoining] technique (e.g. suture, glue) combined transposition of nerves with a nerve autograft |
| 1BN87UH | Excision partial, nerve(s) of forearm and wrist interfascicular split repair [rejoining] technique simple apposition of nerve ends |
| 1BN87UHXXA | Excision partial, nerve(s) of forearm and wrist interfascicular split repair [rejoining] technique nerve autograft (to replace lost length) |
| 1BN87UHXXE | Excision partial, nerve(s) of forearm and wrist interfascicular split repair [rejoining] technique transposition of nerves [e.g. crossover] |
| 1BN87UHXXQ | Excision partial, nerve(s) of forearm and wrist interfascicular split repair [rejoining] technique combined transposition of nerves with a nerve autograft |
| 1BN87WF | Excision partial, nerve(s) of forearm and wrist no rejoining [of nerve ends] nerve end buried |
| **Tonsillectomy** | |
| 1FR87LA | Excision partial, tonsils and adenoids using open (excisional) approach |
| 1FR89LA | Excision total, tonsils and adenoids tonsillectomy alone using device NEC |
| 1FR89LAAK | Excision total, tonsils and adenoids tonsillectomy alone using snare |
| 1FR89WJ | Excision total, tonsils and adenoids tonsillectomy with Adenoidectomy using device NEC |
| 1FR89WJAK | Excision total, tonsils and adenoids tonsillectomy with Adenoidectomy using snare |
| **Shoulder Surgery** | |
| **Implantation, shoulder joint** | |
| 1TA53LAPM | Implantation of internal device, shoulder joint uncemented single-component prosthetic device [e.g. humeral head] |
| 1TA53LAPMA | Implantation of internal device, shoulder joint with bone autograft [uncemented] single-component prosthetic device [e.g. humeral head] |
| 1TA53LAPMK | Implantation of internal device, shoulder joint with bone homograft [uncemented] single-component prosthetic device [e.g. humeral head] |
| 1TA53LAPMN | Implantation of internal device, shoulder joint with synthetic material using single-component prosthetic device [e.g. humeral head] |
| 1TA53LAPMQ | Implantation of internal device, shoulder joint with combined sources of tissue using single-component prosthetic device [e.g. humeral head] |
| 1TA53LAPN | Implantation of internal device, shoulder joint uncemented dual-component prosthetic device [humeral head and glenoid cup] |
| 1TA53LAPNA | Implantation of internal device, shoulder joint with bone autograft [uncemented] dual-component prosthetic device [humeral head and glenoid cup] |
| 1TA53LAPNK | Implantation of internal device, shoulder joint with bone homograft [uncemented] dual-component prosthetic device [humeral head and glenoid cup] |
| 1TA53LAPNN | Implantation of internal device, shoulder joint with synthetic material (e.g. bone paste, cement, Dynagraft, Osteoset) dual component prosthetic device [humeral head and glenoid cup] |
| 1TA53LAPNQ | Implantation of internal device, shoulder joint with combined sources of tissue using dual-component prosthetic device [humeral head and glenoid cup] |
| 1TA53LAPQ | Implantation of internal device, shoulder joint uncemented reverse dual component prosthetic device [humeral cup and glenoid head] |
| 1TA53LAPQA | Implantation of internal device, shoulder joint with bone autograft [uncemented] reverse dual component prosthetic device [humeral cup and glenoid head] |
| 1TA53LAPQK | Implantation of internal device, shoulder joint with bone homograft [uncemented] reverse dual component prosthetic device [humeral cup and glenoid head] |
| 1TA53LAPQN | Implantation of internal device, shoulder joint with synthetic material (e.g. bone paste, cement, Dynagraft, Osteoset) reverse dual component prosthetic device [humeral cup and glenoid head] |
| 1TA53LAPQQ | Implantation of internal device, shoulder joint with combined sources of tissue (e.g. bone graft, cement, paste) reverse dual component prosthetic device [humeral cup and glenoid head] |
| 1TA53LASLN | Implantation of internal device, shoulder joint with synthetic material using cement spacer (temporary) [impregnated with antibiotics] |
| **Repair, shoulder joint** | |
| 1TA80DA | Repair, shoulder joint endoscopic [arthroscopic] approach using simple apposition technique only [e.g. suturing] |
| 1TA80DAAG | Repair, shoulder joint endoscopic [arthroscopic] approach using laser (alone) [to shrink tissue] |
| 1TA80DAFH | Repair, shoulder joint endoscopic [arthroscopic] approach using biodegradable binding device [e.g. Suretac anchor system] |
| 1TA80DAXXA | Repair, shoulder joint endoscopic [arthroscopic] approach using autograft [e.g. bone, interpositional fascia, muscle graft] |
| 1TA80DAXXE | Repair, shoulder joint endoscopic [arthroscopic] approach using local tendon transfer [rebalancing] |
| 1TA80DAXXN | Repair, shoulder joint endoscopic [arthroscopic] approach using synthetic tissue [e.g. mesh, gortex, silastic sheath] |
| 1TA80DAXXQ | Repair, shoulder joint endoscopic [arthroscopic] approach using combined sources of tissue [autograft with synthetic tissue] |
| 1TA80GZ | Repair, shoulder joint endoscopic [arthroscopic] approach using special incisional technique [e.g. multiple burr holes for humeral head revascularization] |
| 1TA80LA | Repair, shoulder joint open approach using simple apposition technique only [e.g. suturing] |
| 1TA80LAFH | Repair, shoulder joint open approach using biodegradable binding device [e.g. Suretac anchor system] |
| 1TA80LAXXA | Repair, shoulder joint open approach using autograft [e.g. bone, interpositional fascia, muscle graft] |
| 1TA80LAXXE | Repair, shoulder joint open approach using local tendon transfer [rebalancing] |
| 1TA80LAXXN | Repair, shoulder joint open approach using synthetic tissue [e.g. mesh, gortex, silastic sheath] |
| 1TA80LAXXQ | Repair, shoulder joint open approach using combined sources of tissue [autograft with synthetic tissue] |
| 1TA80WK | Repair, shoulder joint open approach using special incisional technique [e.g. multiple burr holes for humeral head revascularization] |
| **Extraction, rotator cuff** | |
| 1TC57DA | Extraction, rotator cuff using endoscopic [arthroscopic] approach |
| 1TC57LA | Extraction, rotator cuff using open approach |
| **Destruction, rotator cuff** | |
| 1TC59DA | Destruction, rotator cuff using endoscopic (arthroscopic) approach |
| 1TC59LA | Destruction, rotator cuff using open approach |
| Release, rotator cuff | |
| 1TC72DA | Release, rotator cuff using endoscopic [arthroscopic] approach |
| 1TC72LA | Release, rotator cuff using open approach |
| **Repair, rotator cuff** | |
| 1TC80DA | Repair, rotator cuff endoscopic [arthroscopic] approach using apposition technique [e.g. tendon sutured to tendon] simple repair (without graft or transfer involved) |
| 1TC80DAFH | Repair, rotator cuff using endoscopic [arthroscopic]approach using apposition technique [e.g. tendon sutured to tendon] using biodegradable (binding) device [e.g. biostinger, fastener, anchor, arrow, staple or dart] |
| 1TC80DAXXA | Repair, rotator cuff endoscopic [arthroscopic] approach using apposition technique [e.g. tendon sutured to tendon] with autograft [e.g. tendon, fascia] |
| 1TC80DAXXE | Repair, rotator cuff endoscopic [arthroscopic] approach using apposition technique [e.g. tendon sutured to tendon] with tendon transfer for realignment [e.g. advancement, transposition] |
| 1TC80DAXXK | Repair, rotator cuff endoscopic [arthroscopic] approach using apposition technique [e.g. tendon sutured to tendon] with homograft [e.g. GRAFTJACKET regenerative tissue matrix] |
| 1TC80DAXXN | Repair, rotator cuff endoscopic [arthroscopic] approach using apposition technique [e.g. tendon sutured to tendon] with synthetic tissue [e.g. gortex, mesh] |
| 1TC80DAXXQ | Repair, rotator cuff endoscopic [arthroscopic] approach using apposition technique [e.g. tendon sutured to tendon] with combined sources of tissue [e.g. autograft, tendon transfer, goretex] |
| 1TC80GC | Repair, rotator cuff endoscopic [arthroscopic] approach using tenodesis technique [e.g. tendon looped or sutured to or through bone] simple repair (without graft or transfer involved) |
| 1TC80GCFH | Repair, rotator cuff endoscopic [arthroscopic] approach using tenodesis technique [e.g. tendon looped or sutured to or through bone] using biodegradable (binding) device [e.g. biostinger, fastener, anchor, arrow, staple or dart] |
| 1TC80GCNW | Repair, rotator cuff endoscopic [arthroscopic] approach using tenodesis with screw fixation [e.g. tendon with a bone plug fixed to bone with screw] simple repair (without graft or transfer involved) |
| 1TC80GCNWA | Repair, rotator cuff endoscopic [arthroscopic] approach using tenodesis with screw fixation [e.g. tendon with a bone plug fixed to bone with screw] with autograft [e.g. tendon, fascia] |
| 1TC80GCNWE | Repair, rotator cuff endoscopic [arthroscopic] approach using tenodesis with screw fixation [e.g. tendon with a bone plug fixed to bone with screw] with tendon transfer for realignment[e.g. advancement, transposition] |
| 1TC80GCNWK | Repair, rotator cuff endoscopic [arthroscopic] approach using tenodesis with screw fixation [e.g. tendon with a bone plug fixed to bone with screw] with homograft [e.g. GRAFTJACKET regenerative tissue matrix] |
| 1TC80GCNWN | Repair, rotator cuff endoscopic [arthroscopic] approach using tenodesis with screw fixation [e.g. tendon with a bone plug fixed to bone with screw] with synthetic tissue [e.g. gortex, mesh] |
| 1TC80GCNWQ | Repair, rotator cuff endoscopic [arthroscopic] approach using tenodesis with screw fixation [e.g. tendon with a bone plug fixed to bone with screw] with combined sources of tissue [e.g. autograft, tendon transfer, goretex] |
| 1TC80GCXXA | Repair, rotator cuff endoscopic [arthroscopic] approach using tenodesis technique [e.g. tendon looped or sutured to or through bone] with autograft [e.g. tendon, fascia] |
| 1TC80GCXXE | Repair, rotator cuff endoscopic [arthroscopic] approach using tenodesis technique [e.g. tendon looped or sutured to or through bone] with tendon transfer for realignment[e.g. advancement, transposition] |
| 1TC80GCXXK | Repair, rotator cuff endoscopic [arthroscopic] approach using tenodesis technique [e.g. tendon looped or sutured to or through bone] with homograft [e.g. GRAFTJACKET regenerative tissue matrix] |
| 1TC80GCXXN | Repair, rotator cuff endoscopic [arthroscopic] approach using tenodesis technique [e.g. tendon looped or sutured to or through bone] with synthetic tissue [e.g. gortex, mesh] |
| 1TC80GCXXQ | Repair, rotator cuff endoscopic [arthroscopic] approach using tenodesis technique [e.g. tendon looped or sutured to or through bone] with combined sources of tissue [e.g. autograft, tendon transfer, goretex] |
| 1TC80LA | Repair, rotator cuff open approach using apposition technique [e.g. tendon sutured to tendon] simple repair (without graft or transfer involved) |
| 1TC80LAFH | Repair, rotator cuff open approach using apposition technique [e.g tendon sutured to tendon] using biodegradable (binding) device [e.g biostinger, fastener, anchor, arrow, staple or dart] |
| 1TC80LAXXA | Repair, rotator cuff open approach using apposition technique [e.g. tendon sutured to tendon] with autograft [e.g. tendon, fascia] |
| 1TC80LAXXE | Repair, rotator cuff open approach using apposition technique [e.g. tendon sutured to tendon] with tendon transfer for realignment[e.g. advancement, transposition] |
| 1TC80LAXXK | Repair, rotator cuff using open approach using apposition technique [e.g. tendon sutured to tendon] with homograft [e.g. GRAFTJACKET regenerative tissue matrix] |
| 1TC80LAXXN | Repair, rotator cuff open approach using apposition technique [e.g. tendon sutured to tendon] with synthetic tissue [e.g. gortex, mesh] |
| 1TC80LAXXQ | Repair, rotator cuff open approach using apposition technique [e.g. tendon sutured to tendon] with combined sources of tissue [e.g. autograft, tendon transfer, goretex] |
| 1TC80WU | Repair, rotator cuff open approach using tenodesis technique [e.g. tendon looped or sutured to or through bone] simple repair (without graft or transfer involved) |
| 1TC80WUFH | Repair, rotator cuff open approach using tenodesis technique [e.g. tendon looped or sutured to or through bone] using biodegradable (binding) device [e.g. biostinger, fastener, anchor, arrow, staple or dart] |
| 1TC80WUNW | Repair, rotator cuff open approach using tenodesis with screw fixation [e.g. tendon with a bone plug fixed to bone with screw] simple repair (without graft or transfer involved) |
| 1TC80WUNWA | Repair, rotator cuff open approach using tenodesis with screw fixation [e.g. tendon with a bone plug fixed to bone with screw] with autograft [e.g. tendon, fascia] |
| 1TC80WUNWE | Repair, rotator cuff open approach using tenodesis with screw fixation [e.g. tendon with a bone plug fixed to bone with screw] with tendon transfer for realignment [e.g. advancement, transposition] |
| 1TC80WUNWK | Repair, rotator cuff using open approach using tenodesis with screw fixation [e.g. tendon with a bone plug fixed to bone with screw] with homograft [e.g. GRAFTJACKET regenerative tissue matrix] |
| 1TC80WUNWN | Repair, rotator cuff open approach using tenodesis with screw fixation [e.g. tendon with a bone plug fixed to bone with screw] with synthetic tissue [e.g. gortex, mesh] |
| 1TC80WUNWQ | Repair, rotator cuff open approach using tenodesis with screw fixation [e.g. tendon with a bone plug fixed to bone with screw] with combined sources of tissue [e.g. autograft, tendon transfer, goretex] |
| 1TC80WUXXA | Repair, rotator cuff open approach using tenodesis technique [e.g. tendon looped or sutured to or through bone] with autograft [e.g. tendon, fascia] |
| 1TC80WUXXE | Repair, rotator cuff open approach using tenodesis technique [e.g. tendon looped or sutured to or through bone] with tendon transfer for realignment[e.g. advancement, transposition] |
| 1TC80WUXXK | Repair, rotator cuff open approach using tenodesis technique [e.g. tendon looped or sutured to or through bone] with homograft [e.g. GRAFTJACKET regenerative tissue matrix] |
| 1TC80WUXXN | Repair, rotator cuff open approach using tenodesis technique [e.g. tendon looped or sutured to or through bone] with synthetic tissue [e.g. gortex, mesh] |
| 1TC80WUXXQ | Repair, rotator cuff open approach using tenodesis technique [e.g. tendon looped or sutured to or through bone] with combined sources of tissue [e.g. autograft, tendon transfer, goretex] |
| **Appendectomy** | |
| 1NV89DA | Excision total, appendix using endoscopic [laparoscopic] approach |
| 1NV89LA | Excision total, appendix using open approach |
